# Intentions and actions towards leaving healthcare work since COVID-19: group-based trajectory analyses in the UK-REACH cohort

**DOI:** 10.1177/01410768261419581

**Published:** 2026-02-25

**Authors:** Raman Mishra, Anna L Guyatt, Asta Medisauskaite, Christopher A Martin, Shingai Musuka, Srinivasa Vittal Katikireddi, Katherine Woolf, Manish Pareek, On behalf of the I-CARE Study Collaborative Group

**Affiliations:** 1 University College London Medical School, London, UK; 2 Department of Population Health Sciences, University of Leicester, Leicester, UK; 3 Leicester NIHR Biomedical Research Centre, Leicester, UK; 4 Division of Public Health and Epidemiology, School of Medical Sciences, University of Leicester, Leicester, UK; 5 Department of Infection and HIV Medicine, University Hospitals of Leicester NHS Trust, Leicester, UK; 6 Development Centre for Population Health, University of Leicester, Leicester, UK; 7 School of Nursing and Midwifery, University of Central Lancashire, Preston, UK; 8 MRC/CSO Social and Public Health Sciences Unit, School of Health and Wellbeing, University of Glasgow, UK; 9 NIHR Applied Research Collaboration East Midlands, Leicester, UK; 10 The members of I-CARE Study Collaborative Group are listed in the Acknowledgements

**Keywords:** health policy, ethnic studies, Epidemiologic studies, Epidemiology, Occupational and environmental medicine, Getting and changing jobs (including job descriptions and contracts), Medical careers

## Abstract

**Objectives:**

Retaining healthcare workers is often challenging. We studied predictors and patterns of leaving or wanting to leave healthcare work over time (attrition intentions and/or actions) in a UK cohort.

**Design:**

Using five waves of longitudinal data (2020–2024), we performed group-based trajectory analysis to identify subgroups of healthcare workers with similar patterns of attrition intentions and/or actions over time. We described the characteristics of individuals in each trajectory, and used age-, sex- and occupation-adjusted multinomial logistic regression to identify predictors of trajectory membership.

**Settings:**

United Kingdom.

**Participants:**

Participants of the United Kingdom Research study into Ethnicity and COVID-19 outcomes in Healthcare workers (UK-REACH, *N* = 5499).

**Main outcome measures:**

‘Attrition intentions and/or actions’ was defined as a healthcare worker having intentions (or having taken action) to leave their role (including early retirement).

**Results:**

We identified three trajectories of attrition intentions and/or actions: consistently low (47.7%), moderate and increasing (36.8%) and consistently high (15.5%). Attrition intentions were linked to action: the majority of the ‘consistently high’ group had taken action to leave or change their job. Factors associated with the two latter trajectories included older age, job role (nursing, midwifery and dental), experiencing discrimination and poor mental health. Financial insecurity was strongly associated with attrition intentions and/or actions.

**Conclusions:**

Over half of UK healthcare workers surveyed intended or had taken action to leave or change their role. Interventions to enhance workforce sustainability could be targeted at older workers and those in nursing, midwifery and dental roles and focus on reducing discrimination, improving mental health and protecting the financial security healthcare worker jobs offer.

## Introduction

Many healthcare systems in high-income countries, including the United Kingdom, report difficulties in retaining healthcare staff.^1^ Despite an increase in absolute numbers of staff in the English National Health Service (NHS) in the past 15 years, demand for healthcare services is outstripping supply, with over 111,000 vacancies unfilled as of 2024.^2^ Ongoing increases in training and recruitment will only alleviate pressure on services if the NHS can retain its staff, since retention prevents a vicious cycle of worsening pressure on remaining staff, and increases capacity.^2–4^

Shortfalls in the NHS are predicted to get worse over time, with a workforce gap of 360,000 predicted by 2036/2037.^3^ Shortages are not equal across all staff and demographic groups. Voluntary resignations have trebled in the past decade, with the top reasons for voluntary resignations due to work–life balance or health issues. Previous studies highlighted important roles for poor mental health and well-being, discrimination and workload.^5,6^

There has been a particularly sharp upswing in voluntary resignations across the NHS since 2020–2021.^2^ Previous studies have highlighted factors that predict healthcare workers having intentions or taking action to leave their jobs,^6^ but fewer studies have analysed how individuals’ attrition intentions evolve over time, and drivers of any change.

Using a large longitudinal cohort study of UK healthcare workers, we aimed to: (i) describe changes in attrition intentions and/or actions over time, using five waves of questionnaire data collected between 2020 and 2024; (ii) identify shared patterns of attrition intentions and/or actions over time (‘trajectories’) amongst subgroups of healthcare workers and (iii) investigate demographic, health, occupational and personal factors predicting different trajectories.

## Methods

### Study design and participants

The United Kingdom Research study into Ethnicity and COVID-19 outcomes in Healthcare workers (UK-REACH) cohort has been described previously.^7^ Recruitment of the original cohort took place between December 2020 and March 2021, and included participants over 16 years, living and working in the United Kingdom, who were healthcare workers or ancillary workers in a healthcare setting, or registered with the General Medical Council, Nursing and Midwifery Council, General Dental Council, Health and Care Professions Council, General Optical Council, General Pharmaceutical Council or the Pharmaceutical Society of Northern Ireland.

We used five waves of data starting from December 2020 to March 2024 (Supplementary Methods) from the UK-REACH cohort study. We defined our eligible analysis sample as participants with at least two waves of outcome data (available between waves 2 and 5).

### Description of variables

Our key outcome measure (having attrition intentions and/or actions) was derived from the following question, asked at each of waves 2–5: ‘*Has the COVID-19 pandemic made you consider or act upon leaving your work?*’. Participants stating that they had either thought about or taken action towards leaving their role including taking early retirement were coded as 1, with others coded as 0. Since participants at waves 4 and 5 were only asked this question if they were working, we supplemented the outcome for all waves by also coding as 1 those healthcare staff who stated in another variable that they were currently unemployed or retired from their job (see also Supplementary Methods).

We sought to explain predictors of patterns of attrition intentions and/or actions over time using the following groups of variables, collected at baseline (2020–2021) from the UK-REACH cohort (Supplemental Table 1). The four domains and predictors were defined based upon literature:

1. Demographic characteristics: Age, sex, occupation,^8–10^ ethnicity and migration status.
2. Mental health measures: Depression,^11^ anxiety^11^ and post-traumatic stress disorder (PTSD)^12^.
3. Work characteristics: Clinical workload (number of patients consulted with per week), experiences of discrimination, fairness in the workplace, feeling secure to raise concerns in the workplace and trusting one’s employer to address these concerns; access to personal protective equipment, and redeployment (during the pandemic).
4. Personal circumstances: Loneliness and self-reported financial security.

### Statistical analysis

Using outcome data measured on individuals for at least two waves (to a maximum of four) as input, we used group-based trajectory modelling (GBTM)^13^ to identify subgroups of healthcare staff with similar trajectories of attrition intentions and/or actions levels over time. This method has been previously used in understanding occupational outcomes among the general population.^14^ GBTM uses a full information maximum likelihood (FIML) estimation approach which handles missing data efficiently.^15^ Patterns of missingness across waves are tabulated in Supplemental Table 2. Factors related to non-response over time are described in Supplemental Table 3. The outcome variable attrition intentions and/or actions is binary; therefore, we modelled the GBTM using a logit distribution. More details on the implementation of the GBTM are provided in Supplemental Table 4 which presents the statistical information on the adequacy of group-based trajectory models.

To examine factors associated with the attrition trajectories found, we described the membership of each of the groups in terms of the various demographic, mental health, discrimination and organisational factors mentioned previously. We then conducted multinomial logistic regression model (adjusted for age, sex and occupation) to evaluate the relationship between healthcare workers attrition levels and these same factors, in separate models (Supplemental Table 7). To address missing data in the covariates, we performed imputation and re-ran the regression models with the same adjustments (Supplemental Table 8). As a sensitivity analysis, we fitted an additional model that included all the covariates (Supplemental Table 9).

Data analysis was carried out using Stata version 17 and the ‘traj’ package for GBTM, with statistical code available on github (https://github.com/raman555/Group_Based_Trajectory_Model). We used the Guidelines for Reporting on Group Based Trajectory Studies^16^ to present findings (Supplemental Table 5 for checklist). Missing covariate data were imputed using *mice* package in R (Supplemental Method).

## Results

Our analysis sample comprised 5499 healthcare workers with data across four data collection waves. Sample characteristics are presented in Supplemental Table 3. The analysis sample generally reflected the baseline sample in terms of sex and occupation; however, there were some differences in terms of age and ethnicity, with younger healthcare workers, and Asian healthcare workers being less likely to have complete data for all waves.

Attrition intentions and/or actions increased over time from nearly one in four (29.6%) of healthcare workers intending to leave in 2021 to nearly one in two (47.1%) in 2024 (Supplemental Table 6). Broadly, similar patterns were observed when (i) tabulating outcome data for each wave separately, and (ii) when restricting to participants with complete data for all four outcomes.

**Figure 1.**
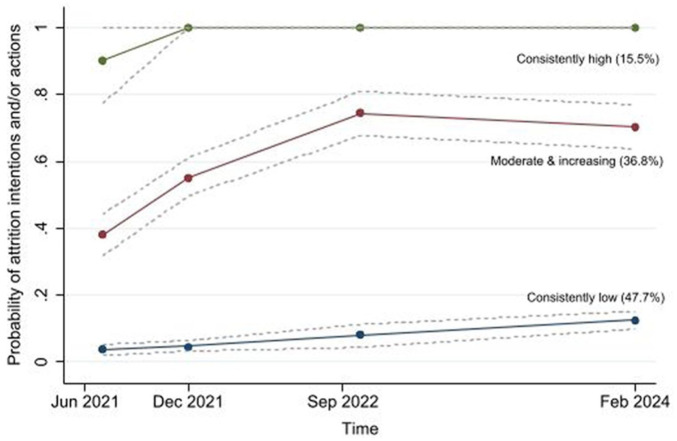
Observed intentions and actions towards leaving healthcare work using group-based trajectory model across four waves. Estimates of the means of the group probabilities over time (with circular dots reflecting estimates for each wave) with 95% confidence intervals are shown. Statistical rationale for selection of the optimal model are presented in Supplemental Table 5. The narrow and consistent width confidence intervals (dashed lines) indicate a good fit of the model to the data.

The optimal trajectory model identified three distinct trajectories of attrition intentions and/or actions (see Figure 1).

– The ‘consistently low’ group (47.7%) showed consistently low attrition intentions and/or actions.
– The ‘moderate and increasing’ group (36.8%) showed an initial increase in attrition from time points 1 to 3, with little change after time point 3.
– The ‘consistently high’ group (15.5%) had consistently high attrition intention and/or actions over time.

Table 1 shows that the members of the ‘consistently high’ trajectory were most likely to have taken action (as opposed to only having intentions) to leave or change their job role, with 21.9% having left or retired by wave 5, and 31.3% having made other changes to their role, such as reducing hours, reducing clinical work, or changing field. The comparable percentages were 12.6% (leaving or retiring) and 22.7% (other changes) in the ‘moderate and increasing group’, and 1.4% (leaving or retiring) and 6.9% (other changes) in the ‘consistently low’ group.

**Table 1.** Proportion of individuals who have taken action to leave their job role at wave 5, by trajectory membership.

Attrition action (*N* = 3466)	Consistently low	Moderate and increasing	Consistently high	Total
No actions*	1554 (91.7%)	691 (64.6%)	328 (46.7%)	2573 (74.2%)
Action taken for leaving job and early retirement	24 (1.4%)	135 (12.6%)	154 (21.9%)	313 (9.0%)
Action taken for reducing working hours, reducing clinical jobs and changing field	117 (6.9%)	243 (22.7%)	220 (31.3%)	580 (16.7%)

* That is, in wave 5, individual had intentions only, or no actions or intentions.

Table 2 shows the group membership across the key characteristics, providing insights into the factors influencing attrition intentions and/or actions. In comparison to those in ‘moderate and increasing’ or ‘consistently low’ trajectory groups, the ‘consistently high’ trajectory group had relatively more staff aged 50–59 years; doctors/medical support and allied health professionals (AHPs); staff meeting criteria for depression, anxiety and PTSD; staff experiencing discrimination, financial difficulties and higher levels of loneliness; staff who reported not having access to personal protective equipment (PPE) at all times in 2020–2021; staff who had been redeployed; staff who felt less secure to raise concerns and who were less trusting that their organisation would address these concerns.

**Table 2.** Membership in each group for attrition according to key characteristics.

Characteristics	Total	Consistently low *n*(%)/*M*(SD)	Moderate and increasing *n*(%)/*M*(SD)	Consistently high *n*(%)/*M*(SD)	*p*-Value
	*N* = 5499	2676 (48.7)	1652 (30.0)	1171 (21.3)	
Age category					
18–29	495 (9.3)	255 (9.9)	159 (10.0)	81 (7.2)	<0.0001
30–39	1180 (22.3)	673 (26.0)	328 (20.6)	179 (15.9)	
40–49	1277 (24.1)	690 (26.7)	346 (21.8)	241 (21.4)	
50–59	1651 (31.1)	657 (25.4)	530 (33.3)	464 (41.3)	
60+ years	698 (13.2)	312 (12.1)	227 (14.3)	159 (14.1)	
Gender					
Male	1313 (24.7)	669 (25.8)	385 (24.1)	259 (23)	0.156
Female	4002 (75.3)	1924 (74.2)	1211 (75.9)	867 (77)	
Occupation					
Doctors and medical support	1237 (23.9)	347 (22.4)	248 (22.9)	642 (25.4)	<0.0001
Nurses, NAs and midwives	1157 (22.4)	371 (23.9)	310 (28.6)	476 (18.8)	
AHPs (not including scientists)	1557 (30.1)	456 (29.4)	281 (25.9)	820 (32.4)	
Pharmacy	97 (1.9)	33 (2.1)	11 (1.0)	53 (2.1)	
Healthcare scientist	249 (4.8)	60 (3.9)	54 (5.0)	135 (5.3)	
Ambulance	178 (3.4)	62 (4.0)	36 (3.3)	80 (3.2)	
Dental	295 (5.7)	101 (6.5)	82 (7.6)	112 (4.4)	
Optical	125 (2.4)	41 (2.6)	26 (2.4)	58 (2.3)	
Administrative	109 (2.1)	31 (2.0)	13 (1.2)	65 (2.6)	
Other	161 (3.1)	50 (3.2)	23 (2.1)	88 (3.5)	
Ethnicity					
White British/Irish	3454 (67.7)	1663 (66.4)	1052 (69.0)	739 (68.9)	<0.001
White other/Gypsy/ Irish Travellers	377 (7.4)	177 (7.1)	115 (7.5)	85 (7.9)	
Asian	821 (16.1)	436 (17.4)	223 (14.6)	162 (15.1)	
Black	157 (3.1)	100 (4.0)	31 (2.0)	26 (2.4)	
Mixed	218 (4.3)	90 (3.6)	77 (5.1)	51 (4.8)	
Other	76 (1.5)	40 (1.6)	26 (1.7)	10 (0.9)	
Born abroad					
Born in United Kingdom	4051 (78.3)	1957 (77.1)	1224 (79.2)	870 (80.0)	0.088
Born outside United Kingdom	1121 (21.7)	582 (22.9)	322 (20.8)	217 (20.0)	
Financial difficulties (mean (SD))	1.7 (0.9)	1.7 (0.9)^a,b^	1.9 (0.9)^a,c^	2.0 (1.0)^b,c^	<0.0001
Depression					
No	4426 (87.5)	2274 (91.4)	1313 (86.9)	839 (79.1)	<0.0001
Yes	633 (12.5)	213 (8.6)	198 (13.1)	222 (20.9)	
Anxiety					
No	4220 (83.0)	2191 (87.8)	1245 (81.7)	784 (73.5)	<0.0001
Yes	864 (17.0)	304 (12.2)	278 (18.3)	282 (26.5)	
PTSD					
No	3392 (66.4)	1841 (73.4)	963 (63.1)	588 (54.9)	<0.0001
Yes	1713 (33.6)	667 (26.6)	562 (36.9)	484 (45.1)	
Patients attended					
Zero	690 (14.1)	375 (15.3)	175 (12.1)	140 (14.1)	<0.0001
Low	1275 (26.1)	682 (27.9)	349 (24.2)	244 (24.5)	
Medium	1323 (27.1)	667 (27.3)	389 (27.0)	267 (26.8)	
High	1594 (32.7)	720 (29.5)	529 (36.7)	345 (34.6)	
Discrimination					
No discrimination	3546 (73.1)	1895 (79.4)	1007 (69.4)	644 (63.5)	<0.0001
From patients	658 (13.6)	270 (11.3)	235 (16.2)	153 (15.1)	
From colleagues	433 (8.9)	156 (6.5)	135 (9.3)	142 (14.0)	
From patients and colleagues	215 (4.4)	66 (2.8)	74 (5.1)	75 (7.4)	
Work fairness (mean (SD))	4.4 (0.8)	4.5 (0.7)^a,b^	4.4 (0.8)^a,c^	4.2 (0.9)^b,c^	<0.0001
Feeling secure to raise concern (mean (SD))	4.3 (0.9)	4.3 (0.9)^a,b^	4.2 (0.9)^a,c^	4.1 (1.0)^b,c^	<0.0001
Trusting employer to address concern (mean (SD))	4.1 (1.0)	4.1 (1.0)^a,b^	3.9 (1.0)^a,c^	3.6 (1.2)^b,c^	<0.0001
Access to PPE					
Not all time	626 (13.6)	244 (10.6)	211 (15.3)	171 (18.2)	<0.0001
All the time	3985 (86.4)	2054 (89.4)	1164 (84.7)	767 (81.8)	
Job redeployment					
No	3630 (79.6)	1813 (81.0)	1095 (80.2)	722 (75.5)	0.002
Yes	930 (20.4)	426 (19.0)	270 (19.8)	234 (24.5)	
Loneliness (mean (SD))	4.6 (1.7)	4.6 (1.7)^a,b^	4.9 (1.9)^a,c^	5.2 (1.9)^b,c^	<0.0001

AHPs: allied health professionals; NAs: nurses/nursing associates; PPE: personal protective equipment; PTSD: post-traumatic stress disorder; SD: standard deviation.

Chi-square statistics is used for categorical variables, and ANOVA is used for scales to compute *p*-values.

a,b,c Post-hoc comparison identifying significant differences between the groups.

The ‘moderate and increasing’ attrition trajectory group had a larger percentage of staff from nurses/nursing associates (NAs)/midwives and dental occupational groups, and those with a higher clinical workload, in comparison to the ‘consistently low’ or ‘consistently high trajectory groups. The ‘consistently low’ group had the lowest proportion of those from Black ethnic groups.

**Figure 2.**
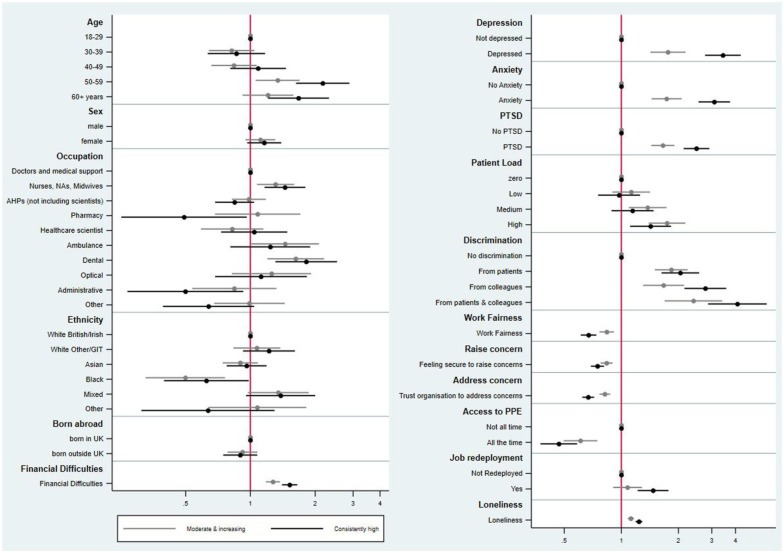
Relative risk ratios (RRRs) and confidence intervals from multinomial logistic regression comparing attrition trajectories. The ‘consistently low’ trajectory group is used as the reference category. The RRR of selecting ‘moderate and increasing’ over the ‘consistently low’ is presented in light grey, whereas the RRR of selecting ‘consistently high’ over ‘consistently low’ is presented in dark grey colour.

Figure 2 presents the relative risk ratio (RRR) from the multinomial logistic regression (adjusted for age, sex and occupational group), comparing the ‘moderate and increasing’ and ‘consistently high’ trajectories to the ‘consistently low’ trajectory (Supplemental Table 7).

Staff were more likely to belong to the ‘moderate and increasing’ (RRR = 1.34, 95%CI: 1.06–1.69) and ‘consistently high’ (RRR = 2.17, 95%CI: 1.63–2.88) trajectories of attrition intentions and/or action, if they were aged 50–59 years rather than 18–29 years.

Compared to doctors and medical support staff, the most striking difference in risk of belonging to the ‘moderate and increasing’ and ‘consistently high attrition’ trajectories was for dental professionals (‘moderate and increasing’ RRR = 1.62, 95%CI: 1.20–2.20; ‘consistently high’ RRR = 1.82, 95%CI:1.31–2.53), and for nurses, NAs, and midwives (moderate and increasing RRR = 1.31, 95%CI:1.07–1.60; ‘consistently high’ RRR = 1.45, 95%CI: 1.16–1.80). When comparing membership of attrition intentions and/or action trajectories by ethnicity, those from Black ethnic background were less likely to belong to the ‘moderate and increasing’ (RRR = 0.50, 95%CI: 0.33–0.76) and ‘consistently high’ (RRR = 0.62, 95%CI: 0.4–0.98) groups compared to those from White background. Notably, being born outside the United Kingdom was not associated with membership of the trajectories.

For healthcare workers meeting screening criteria for anxiety, depression and PTSD, there were profound differences between risk of being in the moderate and increasing and consistently high (compared to low) trajectories of attrition, with healthcare workers generally 1–2 times more likely to be in the moderate and increasing attrition trajectory, and 2–3 times more likely to be in the consistently high trajectory. Loneliness was also a strong predictor of belonging to the two consistently high attrition classes. Another predictor of being in the moderate and increasing and consistently high attrition intention categories were being in a position of relative financial insecurity (RRR [moderate/increasing vs low per point increase on Likert scale] 1.27 95%CI: 1.18–1.37); RRR [high vs low] 1.52, 95%CI: 1.4–1.65).

Experiences of discrimination were associated with membership of the ‘consistently high’ group, with the highest point estimate being for staff who had experienced discrimination from both patients and colleagues, who were 4.09 times as likely to belong to the consistently high trajectory compared to the consistently low (95%CI: 2.87–5.84). Being redeployed during the pandemic was associated with membership of the consistently high attrition trajectory (RRR 1.47, 95%CI: 1.22–1.77).

Some factors were associated with healthcare staff in the consistently high group being less likely to consider or act upon leaving over time, including access to PPE during the height of the pandemic, fairness in the workplace, healthcare workers feeling able to raise concerns with their employer and trusting their employer to address these concerns. Furthermore, healthcare staff reporting the highest clinical workload at baseline (during the pandemic) versus those reporting no patient contact, the likelihood of belonging to the moderate and increasing trajectory was higher than the risk of belonging to the consistently high trajectory, although confidence intervals overlapped.

## Discussion

The findings from this longitudinal study of 5499 healthcare employees provide important insights into the patterns of attrition intentions and/or actions over 4 years during and after the COVID-19 pandemic. We identified three distinct trajectories of attrition intentions and/or actions: ‘consistently low’, ‘moderate and increasing’ and ‘consistently high’, with over 50% of staff belonging to the latter two groups. At wave 5, over half of staff in the ‘consistently high’ attrition group had left or changed their role, compared to a third in the moderate and increasing group and only 7% in the consistently low group, demonstrating the link between intentions and actions.

Some demographic and occupational groups had higher risk for attrition. Workers aged 50 years and older were more likely to belong to the moderate/increasing and consistently high trajectories,^17^ which fits with the observation that these participants would be approaching retirement age, and our primary outcome included those considering/acting upon taking early retirement. In addition to strategies that may be broadly applicable across staff groups, such as those to prevent burnout, this may also suggest a role for targeted retention strategies for older healthcare employees, such as flexible work schedules and retirement planning support.^8,18,19^ We also note that higher proportions of Asian and Black healthcare staff were in the consistently low attrition trajectory in comparison to the White British/Irish^8^ group. Although few studies on ethnicity and healthcare worker attrition exist, our results contrast with a US study which found that ethnic minority healthcare workers were more likely to leave their roles.^20^ Our results underscore the need to understand unique challenges faced by different staff groups: for example, to understand whether lower attrition levels are because some staff truly want to stay in their roles, or conversely, whether this is driven at least in part by a lack of viable alternatives. Possible factors include immigration restrictions for staff who have migrated to the United Kingdom, and other experiences that may reduce job mobility,^21^ such as caring responsibilities, limited opportunities for career advancement and experiences of discrimination which may disempower staff to make the changes they would like to make.

Occupational roles also influenced attrition dynamics, with nurses, midwives and dental staff more likely than those in medical roles to have moderate and consistently high attrition intentions and actions.^9^ Specific growing pressures on these groups have been recognised, including delays in contract reform, underinvestment and private sector growth for dentists^21,22^ and workload pressures, poor work–life balance, development, contractual and financial pressures for nurses.^22^ This contrasts with staff in pharmacy and administrative roles, who were less likely to leave despite similar intentions. These role-specific trends suggest the need for tailored interventions addressing the unique stressors faced by different groups of healthcare workers.

Mental health and financial challenges emerged as key predictors of attrition intentions, and these factors reflect the results of a global systematic review on turnover intentions.^6^ Workers with depression, anxiety and PTSD were highly likely to report moderate and consistently high attrition intentions and/or actions.^9^ We note that financial insecurity was associated with consistently higher attrition,^23^ which could suggest that these healthcare workers may be considering whether leaving their role would provide them with better financial prospects. Indeed, other literature recognises that financial pressures may become so severe that some migrant staff are compelled to leave the United Kingdom for better pay elsewhere.^24^ There are many workplace predictors of well-being, including job content, workload (and work–life balance), autonomy and security; ways in which staff are managed, remunerated and supported (including after experiencing adverse events, such as harassment); plus organisational factors, including the culture and collegiality of the workplace.^6,25^ Thus, initiatives to improve well-being may be multifaceted, noting that external pressures will also affect well-being and may drive differences between socio-demographic groups.^26^

Employees facing discrimination, whether from patients, colleagues or both, are more likely to report consistently high attrition.^27,28^ In addition to the factors contributing to high attrition among healthcare workers, several positive influences may play a role in retaining healthcare staff. Evidence suggests that workers who experience higher levels of workplace fairness, have trust in their employers and have a strong sense of belonging are significantly less likely to leave their roles in healthcare settings.^25^ These findings highlight the need for clearly actionable policies that foster a supportive, inclusive and fair workplace culture.

Our study also revealed a nuanced relationship between workload and attrition. While high levels of patient contact may be associated with increased stress, in this study, workers in contact with more patients were more likely to have moderate and increasing intentions than consistently high intentions. This could suggest that factors beyond workload, such as workplace support, play an important role in attrition decisions. Policies aimed at managing workloads must therefore be complemented by broader organisational reforms to address the underlying causes of dissatisfaction.^29^

This study has several notable strengths. To our knowledge, ours is the first study to explore predictors of trajectories of attrition intentions and/or actions over time in a large population-based cohort, rather than relying on static measures. The questionnaire nature of our study meant we were able to study many factors that are not readily available from administrative data. Additionally, the selection of optimal trajectories in the GBTM analysis was justified using appropriate guidelines and summary statistics.

We acknowledge several limitations that may influence the interpretation of the findings. One of the key methodological considerations relates to the use of data from at least two waves to identify distinct trajectories of attrition intentions and/or actions. Restricting the sample to participants with data from three or more waves would have significantly reduced the sample size, potentially compromising the generalizability of the findings. While the analysis sample is broadly comparable to the baseline cohort for outcome and key exposures, the loss to follow-up between baseline and wave 2 may have introduced selection bias. Although the GBTM approach uses FIML analysis to handle missing data in the outcomes, we acknowledge some differences in predictors (such as age and ethnicity), associated with participation in the cohort over time, and that relate to attrition.

Another limitation pertains to the phrasing of the outcome, which references intentions/actions taken due to the COVID-19 pandemic. However, we think it is reasonable to expect that this measure could also capture broader pressures which may influence healthcare workers’ attrition intentions and/or actions. We also acknowledge that the phrasing of the main question used to define the outcome measure was such that participants may have answered ‘yes’ to having attrition intentions after the first instance of having reported them. However, in practice, we observe that some participants reverted to answering ‘no’ to attrition intentions in later time waves after saying ‘yes’ in earlier ones, which gives us confidence that our measure could capture a reduction in intentions over time, even if our returned trajectories did not capture this. We studied attrition intentions and actions together as we would have been underpowered to study actions separately. However, as stated above, we note that those with in the consistently high group were most likely to have taken action by wave 5, suggesting a possible link between intentions and actions. Although some outcome data were missing at this wave, distribution was generally consistent across waves. Finally, we acknowledge that the date of our last data collection (February 2024) predates several UK events, which may affect retention of staff from ethnic minority and migrant groups including hate-based rioting in the United Kingdom, and introduction of visa rules restricting healthcare workers’ rights to bring dependants with them to the United Kingdom. Future efforts are underway in UK-REACH to collect a sixth wave of longitudinal data which will be valuable for exploring the impact of these context further.

## Conclusion

In conclusion, this study highlights the demographic, occupational and workplace factors predicting trajectories of attrition intentions and/or actions among healthcare staff. Understanding modifiable occupational drivers of poor workplace well-being, including factors that may be experienced disproportionately by minoritised groups, are likely to be important strategies for reducing staff attrition intentions and/or actions. Nuances in predictors of retention are also important to understand: it cannot be assumed that staff who remain in post are content; negative factors that keep people in their posts even if they are unhappy will also be important to study in future work, and may vary by socio-demographic groups and affect well-being and productivity. Ultimately, our results suggest that strategies to improve retention and reduce attrition could focus on older workers and those in clinical roles and should aim to reduce discrimination, improve mental health and protect the financial security of healthcare workers. Together, these are likely to support the delivery of quality healthcare, safeguard employee well-being and promote long-term system resilience.

## Supplemental Material

sj-docx-1-jrs-10.1177_01410768261419581 – Supplemental material for Intentions and actions towards leaving healthcare work since COVID-19: group-based trajectory analyses in the UK-REACH cohort Supplemental material, sj-docx-1-jrs-10.1177_01410768261419581 for Intentions and actions towards leaving healthcare work since COVID-19: group-based trajectory analyses in the UK-REACH cohort by Raman Mishra, Anna L Guyatt, Asta Medisauskaite, Christopher A Martin, Shingai Musuka, Srinivasa Vittal Katikireddi, Katherine Woolf and Manish Pareek in Journal of the Royal Society of Medicine

sj-docx-2-jrs-10.1177_01410768261419581 – Supplemental material for Intentions and actions towards leaving healthcare work since COVID-19: group-based trajectory analyses in the UK-REACH cohort Supplemental material, sj-docx-2-jrs-10.1177_01410768261419581 for Intentions and actions towards leaving healthcare work since COVID-19: group-based trajectory analyses in the UK-REACH cohort by Raman Mishra, Anna L Guyatt, Asta Medisauskaite, Christopher A Martin, Shingai Musuka, Srinivasa Vittal Katikireddi, Katherine Woolf and Manish Pareek in Journal of the Royal Society of Medicine

## Declarations

**I-CARE study collaborative group:** Manish Pareek (University of Leicester, Chief investigator), Katherine Woolf (University College London, Chief investigator), Carol Woodhams (University of Surrey), Sorin Krammer (University of Surrey), Carol Rivas (University College London), Anna L. Guyatt (University of Leicester), Asta Medisauskaite (University College London), Srinivasa Vittal Katikireddi (University of Glasgow), Padmasayee Papineni (London North West University Healthcare NHS Trust), Susie Lagrata (University College London Hospitals NHS Foundation Trust), Mehrunisha Suleman (University of Oxford), Asad Masood (University of Leicester), Luke Bryant (University of Leicester), Kirandip Gill (PPI contributor), Christopher A Martin (University of Leicester), Mayuri Gogoi (University of Leicester), Nicola Burton-Brown (University of Surrey), Raman Mishra (UCL), Rebecca Moss (UCL), Holly Reilly (University of Leicester) and Vishant Modhwadia (University of Leicester).

**Ethics approval:** The UK-REACH study was reviewed by London - Brighton and Sussex NHS REC. A favourable outcome was received on 12th October 2020, with Work package 2- the longitudinal Questionnaire cohort study - added by substantial amendment with a Favourable outcome received on 20th November 2020. The I-CARE Work package 3 sub-study (including informed consent processes for existing cohort participants and new recruitment) was added via substantial amendment 18, receiving favourable opinion on the 20th August 2024. IRAS project ID: 288316, REC reference: 20/HRA/4718.

**Guarantor:** MP

**Contributorship:** MP conceived of the idea for the original UK-REACH study and led the funding application with KW and ALG. MP and KW conceived of the idea for I-CARE and led the funding application, with CAM, AM, ALG and SVK. The questionnaire was designed by CAM, AM, ALG, KW, MP and the study collaborative group. RM, ALG, AM and SVK formulated the idea for the analysis and contributed to the analysis plan with input from KW and MP. RM analysed the data with input from ALG, AM and SVK. RM and ALG have accessed and verified the underlying data. RM, ALG, and AM drafted the manuscript with input from SVK, KW and MP. RM, ALG, AM, CAM, SM, SVK, KW and MP edited and approved the final version of the manuscript for publication.

**Provenance:** Not commissioned; peer-reviewed by Sun Jae Park and Julie Morris.
