# Supplementary material for: Intentions and actions towards leaving healthcare work since COVID-19: group-based trajectory analyses in the UK-REACH cohort
Source: J R Soc Med. 2026 Feb 25;119(3):73–82. doi: 10.1177/01410768261419581 (PMC12935585; doi:10.1177/01410768261419581)
Supplement: sj-docx-2-jrs-10.1177_01410768261419581 – Supplemental material for Intentions and actions towards leaving healthcare work since COVID-19: group-based trajectory analyses in the UK-REACH cohort [file sj-docx-2-jrs-10.1177_01410768261419581.docx]

Supplementary Material

# Supplementary Methods

## Dates of questionnaire deployment by wave in the UK-REACH cohort study

- Wave 1: December 2020-March 2021
- Wave 2: April 2021-June 2021
- Wave 3: October 2021-December 2021
- Wave 4: June 2022-September 2022
- Wave 5: October 2023 - February 2024

## Additional detail on coding of outcome measures

The outcome measure ‘attrition intentions and/or actions’ was not measured at baseline (wave 1); information was available from waves 2 to 5 only. Participants in waves 4 and 5 were only asked about their attrition intentions and/or actions if they were working, whereas in waves 2 and 3 this information was collected from all the participants, regardless of current working status. Thus, we supplemented the outcome for wave 4 and wave 5 by using additional data provided by participants who stated in another variable that they were currently unemployed or retired. Specifically, we used responses to the following statement: “Please indicate the reason(s) you are not working now”: participants who stated that they were not currently working, and those who answered that they had retired or were currently unemployed were additionally coded as ‘1’ for our attrition intentions and/or actions outcome for that wave.

## Handling of missing data

Missing data were imputed using multiple imputations by chained equations (MICE) in R. The missing data percentage in the analysis sample varies from 3% for sex to 19% for work fairness variable. We assumed missing at random (MAR), conditional on observed covariates. Ten imputed datasets were created, with five iterations per imputation chain. Continuous variables were imputed using predictive mean matching, binary categorical variables with logistic regression, unordered categorical variables with polytomous logistic regression, and ordered categorical variables with proportional odds regression. A predictor matrix was specified to ensure all relevant covariates contributed to the imputation models. Following imputation, multinomial logistic regression models were fitted across each dataset, and estimates were pooled using Rubin’s rules to obtain final coefficients and 95% confidence intervals (Supplementary Table 8).

# Supplementary Tables

**Supplementary Table 1: Description of measures used in analyses**

| **Variable** | **Measure** | **Number of items, and scoring system** | **Diagnostic** |
| --- | --- | --- | --- |
| **Age** | - | 1 item/  5 categories: 18-29 years, 30-39 years, 40-49 years, 50-59 years, and ≥60 years. | - |
| **Sex** | - | 1 item/  2 categories: males and females | - |
| **Occupation** | - | 1 item/  10 healthcare staff groups: Doctors and medical support; Nurses, Nurse Associates (NAs), Midwives; Allied Health Professionals (AHPs) (not including scientists); Pharmacy; Healthcare scientists; Ambulance; Dental; Optical; Administrative staff; Other | - |
| **Ethnicity** | Based on UK National Census | 1 item/  6 groups: White British/Irish, White Other/ Gypsy or Irish Traveller, Asian, Black, Mixed, and Other. | - |
| **Depression** | Patient Health Questionnaire-2 | 2 items/  0 (“not at all”) to 3 (“nearly every day”) | 3 or more suggests depression |
| **Anxiety** | Generalized Anxiety Disorder-2 | 2 items/  0 (“not at all”) to 3 (“nearly every day”) | 3 or more suggests anxiety |
| **Post-traumatic stress disorder (PTSD)** | PTSD Checklist – 2-item Civilian Version | 2 items/  0 (“not at all”) to 4 (“extremely”) | 4 or more suggests PTSD |
| **Patient load per week** | - | 1 item/  3 categories: High - “than 50 patients”; Medium - “21-50 patients”; Low – “1-20 patients”, Zero – “not attending any patients” | - |
| **Discrimination** | - | 4 items/  Faced discrimination in the past 6 months (“yes” or “no”) and if yes, whether this discrimination is from patients, colleagues or from both. | - |
| **Feeling secure to raise concern about unsafe clinical practise** | - | 1 item/  1 (“strongly disagree”) to 5 (“strongly agree”) | - |
| **Trusting employer to address concern** | - | 1 item/  1 (“strongly disagree”) to 5 (“strongly agree”) | - |
| **Access to personal protective equipment** | - | 1 item/  0 (“Not all the time”) to 1 (“Yes, all the time”) | - |
| **Redeployment** | - | 1 item/  “Yes” and “no” | - |
| **Loneliness** | the University of California, Los Angeles (UCLA) loneliness 3 item scale | 3 items/  1 (“Hardly ever”) to 3 (“Often”) | - |
| **Financial difficulties** | - | 1 item/  1 (“Not at all worried”) to 5 (“Extremely worried”) | - |

**Supplementary Table 2: Patterns of missing data for the outcome variable across waves 2-5.**

| **Waves** | **Number of participants with outcome data at these waves^1^** | **Number of participants with any variable at these waves^2^** | **Percentage with missing data for outcome at these waves^3^** |
| --- | --- | --- | --- |
| Wave 1 | NA | 15,117 | NA |
| Wave 2 | 5246 | 5633 | 6.9% |
| Wave 3 | 6195 | 6535 | 5.2% |
| Wave 4 | 3911 | 4246 | 8.0% |
| Wave 5 | 3793 | 4133 | 8.2% |
| At least 1 wave | 8916 | 9388 | 5.0% |
| Waves 2 & 3 | 3637 | 3932 | 9.2% |
| Waves 2 & 4 | 2429 | 2684 | 9.0% |
| Waves 2 & 5 | 2384 | 2664 | 8.9% |
| Waves 3 & 4 | 2974 | 3248 | 9.2% |
| Waves 3 & 5 | 2773 | 3049 | 9.1% |
| Waves 4 & 5 | 2318 | 2621 | 8.8% |
| At least 2 Waves* | 5499* | 5915* | 7.0%* |
| Waves 2 & 3 & 4 | 2110 | 2344 | 9.98% |
| Waves 2 & 3 & 5 | 2048 | 2290 | 11.8% |
| Waves 3 & 4 & 5 | 1981 | 2238 | 11.5% |
| At least 3 Waves | 3174 | 3449 | 7.97% |
| All Waves | 1556 | 1795 | 13.3% |

^1^The outcome measure was not measured at wave 1 (baseline, N=15,117).

^2^This is the total number of individuals with any variable present for the given combination of waves, even if the outcome was missing.

^3^The percentage missing, based on the proportion of the numbers in the first two columns

*Ultimately, we included individuals with at least two outcome measures between waves 2 and 5 in group-based trajectory analysis (N=5,499, max N=5,326 in regression modelling).

**Supplementary Table 3: Participants characteristics by different samples.**

| **Characteristics** | **Missing outcome for all waves** | **Complete at least one wave** | **Complete for at least two waves** | **Complete for all waves** | **Baseline Sample** |
| --- | --- | --- | --- | --- | --- |
|  | **N (%)/ Mean (SD)** | **N (%)/ Mean (SD)** | **N (%)/ Mean (SD)** | **N (%)/ Mean (SD)** | **N (%)/ Mean (SD)** |
| **Sample Size** | 6931 | 8916 | 5499 | 1556 | 15117 |
| **Age Category** |  |  |  |  |  |
| 18-29 | 1052 (15.5) | 908 (11.0) | 495 (9.3) | 96 (6.2) | 1960 (13.0) |
| 30-39 | 1730 (25.4) | 1934 (23.5) | 1180 (22.3) | 285 (18.3) | 3664 (24.4) |
| 40-49 | 1798 (26.4) | 2022 (24.6) | 1277 (24.1) | 394 (25.3) | 3820 (25.4) |
| 50-59 | 1564 (23.0) | 2373 (28.8) | 1651 (31.1) | 528 (34.0) | 3937 (26.2) |
| 60+ years | 656 (9.6) | 993 (12.1) | 698 (13.2) | 252 (16.2) | 1649 (11.0) |
| **Sex** |  |  |  |  |  |
| Male | 1742 (25.5) | 1968 (23.8) | 1313 (24.7) | 427 (27.5) | 3710 (24.6) |
| Female | 5077 (74.5) | 6284 (76.2) | 4002 (75.3) | 1128 (72.5) | 11361 (75.4) |
| **Occupation** |  |  |  |  |  |
| Doctors and medical support | 1622 (25.4) | 1882 (23.6) | 1237 (23.9) | 394 (26) | 3504 (24.4) |
| Nurses, NAs, Midwives | 1279 (20.0) | 1761 (22.0) | 1157 (22.4) | 332 (21.9) | 3040 (21.1) |
| AHPs (not including scientists) | 1940 (30.3) | 2440 (30.5) | 1557 (30.1) | 440 (29.0) | 4380 (30.5) |
| Pharmacy | 138 (2.2) | 159 (2.0) | 97 (1.9) | 35 (2.3) | 297 (2.1) |
| Healthcare scientist | 250 (3.9) | 373 (4.7) | 249 (4.8) | 84 (5.5) | 623 (4.3) |
| Ambulance | 240 (3.8) | 296 (3.7) | 178 (3.4) | 48 (3.2) | 536 (3.7) |
| Dental | 483 (7.6) | 443 (5.5) | 295 (5.7) | 82 (5.4) | 926 (6.4) |
| Optical | 138 (2.2) | 197 (2.5) | 125 (2.4) | 31 (2.0) | 335 (2.3) |
| Administrative | 93 (1.5) | 170 (2.1) | 109 (2.1) | 26 (1.7) | 263 (1.8) |
| Other | 210 (3.3) | 267 (3.3) | 161 (3.1) | 44 (2.9) | 477 (3.3) |
| **Ethnicity** |  |  |  |  |  |
| White British/Irish | 3076 (56.4) | 5155 (66) | 3454 (67.7) | 1082 (72.2) | 8231 (62.1) |
| White Other/GIT | 397 (7.3) | 612 (7.8) | 377 (7.4) | 93 (6.2) | 1009 (7.6) |
| Asian | 1290 (23.7) | 1303 (16.7) | 821 (16.1) | 219 (14.6) | 2593 (19.6) |
| Black | 299 (5.5) | 286 (3.7) | 157 (3.1) | 37 (2.5) | 585 (4.4) |
| Mixed | 235 (4.3) | 317 (4.1) | 218 (4.3) | 54 (3.6) | 552 (4.2) |
| Other | 157 (2.9) | 133 (1.7) | 76 (1.5) | 13 (0.9) | 290 (2.2) |
| **Born abroad** |  |  |  |  |  |
| born in UK | 3774 (67.8) | 6042 (76.4) | 4051 (78.3) | 1260 (82.4) | 9816 (72.9) |
| born outside UK | 1791 (32.2) | 1864 (23.6) | 1121 (21.7) | 269 (17.6) | 3655 (27.1) |
| **Financial Difficulties** | 2.0 (1.0) | 1.9 (0.9) | 1.8 (0.9) | 1.7 (0.9) | 1.9 (1.0) |
| **Depression** |  |  |  |  |  |
| No | 4177 (84.0) | 6645 (86.6) | 4426 (87.5) | 1360 (89.9) | 10822 (85.6) |
| Yes | 794 (16.0) | 1025 (13.4) | 633 (12.5) | 153 (10.1) | 1819 (14.4) |
| **Anxiety** |  |  |  |  |  |
| No | 3945 (78.8) | 6333 (82.1) | 4220 (83.0) | 1289 (84.7) | 10278 (80.8) |
| Yes | 1063 (21.2) | 1379 (17.9) | 864 (17.0) | 232 (15.3) | 2442 (19.2) |
| **PTSD** |  |  |  |  |  |
| No | 2967 (59.0) | 5057 (65.3) | 3392 (66.4) | 1063 (69.9) | 8024 (62.8) |
| Yes | 2062 (41.0) | 2683 (34.7) | 1713 (33.6) | 458 (30.1) | 4745 (37.2) |
| **Patients attended** |  |  |  |  |  |
| zero | 593 (10.3) | 997 (13.3) | 690 (14.1) | 212 (14.8) | 1590 (12.0) |
| Low | 1248 (21.7) | 1921 (25.6) | 1275 (26.1) | 391 (27.2) | 3169 (23.9) |
| Medium | 1673 (29.1) | 2048 (27.3) | 1323 (27.1) | 396 (27.6) | 3721 (28.1) |
| High | 2238 (38.9) | 2525 (33.7) | 1594 (32.7) | 438 (30.5) | 4763 (36.0) |
| **Discrimination** |  |  |  |  |  |
| No discrimination | 3210 (67.5) | 5278 (71.9) | 3546 (73.1) | 1101 (76.4) | 8488 (70.2) |
| From patients | 730 (15.3) | 1025 (14.0) | 658 (13.6) | 181 (12.6) | 1755 (14.5) |
| From colleagues | 511 (10.7) | 689 (9.4) | 433 (8.9) | 106 (7.4) | 1200 (9.9) |
| From patients and colleagues | 308 (6.5) | 347 (4.7) | 215 (4.4) | 53 (3.7) | 655 (5.4) |
| **Work fairness** | 4.3 (0.9) | 4.4 (0.8) | 4.4 (0.8) | 4.4 (0.8) | 4.4 (0.9) |
| **Feeling secure to raise concern** | 4.2 (1.0) | 4.2 (0.9) | 4.2 (0.9) | 4.3 (0.9) | 4.2 (1.0) |
| **Trusting employer to address concern** | 3.9 (1.1) | 3.9 (1.1) | 3.9 (1.0) | 3.9 (1.0) | 3.9 (1.1) |
| **Access to PPE** |  |  |  |  |  |
| Not all time | 918 (17.7) | 1057 (15.0) | 626 (13.6) | 169 (12.4) | 1975 (16.1) |
| All the time | 4266 (82.3) | 6011 (85.0) | 3985 (86.4) | 1199 (87.6) | 10277 (83.9) |
| **Job redeployment** |  |  |  |  |  |
| No | 3727 (79.7) | 5495 (79.6) | 3630 (79.6) | 1069 (78.0) | 9222 (79.7) |
| Yes | 949 (20.3) | 1406 (20.4) | 930 (20.4) | 301 (22.0) | 2355 (20.3) |
| **Loneliness** | 5.0 (1.9) | 4.9 (1.8) | 4.8 (1.8) | 4.7 (1.8) | 4.9 (1.9) |

## Criteria for assessing model fit in group-based trajectory modelling

**Supplementary Table 4: General Statistical Information of Group Based Trajectory Modeling (GBTM)**

| **Shape of trajectory** | **Group numbers** | **BIC** | **Entropy** | **Average posterior probabilities** | **Odds of correct classification** | **Sample size of each group** |
| --- | --- | --- | --- | --- | --- | --- |
| linear | 2 | -8928.87 | 0.69 | 0.94 | 12.62 | 3018 |
|  |  |  |  | 0.88 | 8.71 | 2481 |
| linear | 3 | -8885.29 | 0.71 | 0.91 | 6.62 | 3258 |
|  |  |  |  | 0.53 | 8.59 | 636 |
|  |  |  |  | 0.88 | 17.11 | 1605 |
| linear | 4 | -8869.45 | 0.55 | 0.80 | 11.94 | 1384 |
|  |  |  |  | 0.70 | 8.04 | 1219 |
|  |  |  |  | 0.63 | 15.27 | 542 |
|  |  |  |  | 0.64 | 2.40 | 2354 |
| quadratic | 2 | -8863.53 | 0.70 | 0.93 | 11.33 | 3070 |
|  |  |  |  | 0.88 | 9.64 | 2429 |
| **quadratic** | **3** | **-8852.17** | **0.60** | **0.88** | **7.64** | **2676** |
|  |  |  |  | **0.78** | **8.42** | **1652** |
|  |  |  |  | **0.65** | **6.89** | **1171** |
| cubic | 2 | -8969.94 | 0.68 | 0.92 | 10.65 | 3070 |
|  |  |  |  | 0.91 | 10.66 | 2429 |
| cubic | 3 | -8865.04 | 0.60 | 0.88 | 7.71 | 2676 |
|  |  |  |  | 0.78 | 8.16 | 1652 |
|  |  |  |  | 0.65 | 6.91 | 1171 |

## Statistical methods for group-based trajectory modelling (GBTM)

Group-based trajectory modeling (GBTM) is a statistical method that identifies groups of individuals with similar patterns in a longitudinal measure. We used different combinations of groups and polynomial functions to identify the optimal groups. To estimate the longitudinal trajectories for attrition as a function of time we test the different models from two to four groups and polynomial functions from first order (linear) to third order (Cubic). The selection of the optimal subgroups and the order of the polynomial function used to shape the model is primarily based on Bayesian Information Criterion (BIC), which serves as the most critical criterion in determining model fit as per The Guidelines for Reporting on Latent Trajectory Studies (GRoLTS)^1, 2^. We selected the optimal group having with highest value (least negative) of BIC. After selecting the optimal number of subgroups the model adequacy was assessed using four criteria: i) the average posterior probabilities (APP) are greater than 0.7 ensuring reliable group membership; ii) the odds of correct classification (OCC) for all groups is >0.5, which shows the model’s ability to assign individuals to their respective trajectories accurately; iii) the consistent width and narrowness of 95% Confidence Interval for group membership probabilities; and iv) the proportion of sample size in each group is greater than 5%^3-5^.

**Supplementary Table 5: Checklist: Guidelines for Reporting on Group Based Trajectory Studies**

| **Sr. No.** | **Checklist Item** | **Reported?** |
| --- | --- | --- |
| **1** | Is the metric of time used in the statistical model reported? | Yes (Supplementary table 1) |
| **2** | Is information presented about the mean and variance of time within a wave? | Yes (We have specified the time periods) |
| **3a** | Is the missing data mechanism reported? | Yes (Supplementary table 3) |
| **3b** | Is a description provided of what variables are related to attrition/missing data? | Yes (Supplementary table 4) |
| **3c** | Is a description provided of how missing data in the analyses were dealt with? | Yes (Statistical analysis) |
| **4** | Is information about the distribution of the observed variables included? | Yes (Supplementary Table 4) |
| **5** | Is the software mentioned? | Yes (Statistical analysis) |
| **6a** | Are alternative specifications of within-class heterogeneity considered (e.g., LGCA vs. LGMM) and clearly documented? If not, was sufficient justification provided as to eliminate certain specifications from consideration | No (GBTM assumes that there is no variation between individuals in the same class) |
| **6b** | Are alternative specifications of the between-class differences in variance–covariance matrix structure considered and clearly documented? If not, was sufficient justification provided as to eliminate certain specifications from consideration? | No, the software doesn’t have this function to compute variance-covariance matrix. |
| **7** | Are alternative shape/functional forms of the trajectories described? | Yes (Supplementary Table 6) |
| **8** | If covariates have been used, can analyses still be replicated? | Not relevant – no covariates used |
| **9** | Is information reported about the number of random start values and final iterations included? | Not required, in GBTM start matrix function is specified to override default start values. |
| **10** | Are the model comparison (and selection) tools described from a statistical perspective? | Yes (Statistical analysis) |
| **11** | Are the total number of fitted models reported, including a one-class solution? | Yes (One class solution was not executed. Total number of fitted models are tabulated in Supplementary Table 5) |
| **12** | Are the number of cases per class reported for each model (absolute sample size, or proportion)? | Yes (Supplementary Table 6) |
| **13** | If classification of cases in a trajectory is the goal, is entropy reported? | Yes (Supplementary Table 6) |
| **14a** | Is a plot included with the estimated mean trajectories of the final solution? | Yes (Figure 1) |
| **14b** | Are plots included with the estimated mean trajectories for each model? | No (we have only included the plot for selected model) |
| **14c** | Is a plot included of the combination of estimated means of the final model and the observed individual trajectories split out for each latent class? | No (Not applicable for binary variable) |
| **15** | Are characteristics of the final class solution numerically described (i.e., means, SD/SE, n, CI, etc.)? | Yes (Figure 1) |
| **16** | Are the syntax files available (either in the appendix, supplementary materials, or from the authors)? | Yes (Supplementary file) |

**Supplementary Table 6**: **Attrition intentions and/or actions across outcome waves in different subsets of UK-REACH participants**

| Attrition | Wave 2 | Wave 3** | Wave 4 | Wave 5 |
| --- | --- | --- | --- | --- |
|  | Jul-21 | Dec-21 | Oct-22 | Feb-24 |
| Participants with outcome data available in at least two waves (N=5,499) * | 29.6%  (N=4,145) | 37.9%  (N=4,801) | 46.7%  (N=3,483) | 47.1%  (N=3,299) |
| Participants with outcome data available at each wave separately | 29.3%  (N=5,246) | 38.1%  (N=6,195) | 47.1%  (N=3,911) | 47.5%  (N=3,793) |
| Participants with outcome data available for all four waves (N=1,556) | 28.0%  (N=1,556) | 37.5%  (N=1,556) | 45.6%  (N=1,556) | 46.3%  (N=1,556) |

*Subset of individuals entering group-based trajectory analysis

**Supplementary Table 7: Relative risk ratios (95% CI) from multinomial logistic regression comparing membership of being in the very low attrition intention group compared to other group associated with covariates.**

| **Characteristics (N)** | **Consistently Low** | **Moderate & increasing** | **P-value** | **Consistently high** | **P-value** |
| --- | --- | --- | --- | --- | --- |
| **Age Category** (5132) |  |  |  |  |  |
| 18-29 | (Reference) | (Reference) |  | (Reference) |  |
| 30-39 |  | 0.82 (0.64 - 1.04) | 0.11 | 0.86 (0.63 - 1.17) | 0.34 |
| 40-49 |  | 0.84 (0.66 - 1.07) | 0.15 | 1.09 (0.81 - 1.46) | 0.59 |
| 50-59 |  | 1.34 (1.06 - 1.69) | 0.02 | 2.17 (1.63 - 2.88) | <0.0001 |
| 60+ years |  | 1.21 (0.92 - 1.58) | 0.18 | 1.67 (1.21 - 2.32) | <0.0001 |
| **Gender** |  |  |  |  |  |
| Male | (Reference) | (Reference) |  | (Reference) |  |
| Female |  | 1.11 (0.95 - 1.3) | 0.18 | 1.16 (0.97 - 1.39) | 0.11 |
| **Occupation** |  |  |  |  |  |
| Doctors and medical support | (Reference) | (Reference) |  | (Reference) |  |
| Nurses, NAs, Midwives |  | 1.31 (1.07 - 1.6) | 0.01 | 1.45 (1.16 - 1.8) | <0.0001 |
| AHPs (not including scientists) |  | 0.98 (0.82 - 1.18) | 0.86 | 0.84 (0.69 - 1.04) | 0.11 |
| Pharmacy |  | 1.08 (0.68 - 1.71) | 0.74 | 0.49 (0.25 - 0.96) | 0.04 |
| Healthcare scientist |  | 0.82 (0.59 - 1.15) | 0.25 | 1.04 (0.73 - 1.48) | 0.82 |
| Ambulance |  | 1.45 (1.01 - 2.08) | 0.05 | 1.24 (0.81 - 1.9) | 0.33 |
| Dental |  | 1.62 (1.2 - 2.2) | <0.0001 | 1.82 (1.31 - 2.53) | <0.0001 |
| Optical |  | 1.25 (0.82 - 1.92) | 0.30 | 1.12 (0.69 - 1.83) | 0.65 |
| Administrative |  | 0.84 (0.54 - 1.32) | 0.45 | 0.5 (0.27 - 0.93) | 0.03 |
| Other |  | 0.99 (0.68 - 1.44) | 0.96 | 0.64 (0.39 - 1.04) | 0.07 |
| **Ethnicity** (4933) |  |  |  |  |  |
| White British/Irish | (Reference) | (Reference) |  | (Reference) |  |
| White Other/GIT |  | 1.07 (0.83 - 1.38) | 0.59 | 1.22 (0.92 - 1.61) | 0.16 |
| Asian |  | 0.90 (0.74 - 1.08) | 0.26 | 0.96 (0.78 - 1.19) | 0.71 |
| Black |  | 0.50 (0.33 - 0.76) | <0.0001 | 0.62 (0.4 - 0.98) | 0.04 |
| Mixed |  | 1.35 (0.97 - 1.87) | 0.07 | 1.38 (0.96 - 2) | 0.09 |
| Other |  | 1.08 (0.64 - 1.82) | 0.78 | 0.63 (0.31 - 1.29) | 0.21 |
| **Born abroad** (4994) |  |  |  |  |  |
| Born in UK | (Reference) | (Reference) |  | (Reference) |  |
| Born outside UK |  | 0.92 (0.78 - 1.08) | 0.30 | 0.9 (0.75 - 1.08) | 0.24 |
| **Financial Difficulties** (per point increase) (4928) |  | 1.27 (1.18 - 1.37) | <0.0001 | 1.52 (1.4 - 1.65) | <0.0001 |
| **Depression** (4890) |  |  |  |  |  |
| No | (Reference) | (Reference) |  | (Reference) |  |
| Yes |  | 1.76 (1.42 - 2.18) | <0.0001 | 3.43 (2.77 - 4.26) | <0.0001 |
| **Anxiety** (4913) |  |  |  |  |  |
| No | (Reference) | (Reference) |  | (Reference) |  |
| Yes |  | 1.73 (1.44 - 2.08) | <0.0001 | 3.09 (2.55 - 3.74) | <0.0001 |
| **PTSD** (4932) |  |  |  |  |  |
| No | (Reference) | (Reference) |  | (Reference) |  |
| Yes |  | 1.66 (1.44 - 1.91) | <0.0001 | 2.49 (2.13 - 2.91) | <0.0001 |
| **Patients attended** (4717) |  |  |  |  |  |
| Zero | (Reference) | (Reference) |  | (Reference) |  |
| Low |  | 1.13 (0.9 - 1.42) | 0.30 | 0.97 (0.75 - 1.25) | 0.83 |
| Medium |  | 1.38 (1.09 - 1.73) | 0.01 | 1.15 (0.89 - 1.48) | 0.30 |
| High |  | 1.74 (1.39 - 2.18) | <0.0001 | 1.43 (1.11 - 1.83) | 0.01 |
| **Discrimination** (4686) |  |  |  |  |  |
| No discrimination | (Reference) | (Reference) |  | (Reference) |  |
| From patients |  | 1.83 (1.5 - 2.24) | <0.0001 | 2.05 (1.63 - 2.57) | <0.0001 |
| From colleagues |  | 1.67 (1.3 - 2.14) | <0.0001 | 2.77 (2.15 - 3.57) | <0.0001 |
| From patients and colleagues |  | 2.4 (1.69 - 3.41) | <0.0001 | 4.09 (2.87 - 5.84) | <0.0001 |
| **Work Fairness** (per point increase) (4467) |  | 0.84 (0.77-0.92) | <0.0001 | 0.68 (0.61-0.74) | <0.0001 |
| **Feeling secure to raise concern** (per point increase) (4634) |  | 0.84 (0.78 - 0.9) | <0.0001 | 0.75 (0.69 - 0.81) | <0.0001 |
| **Trusting employer to address concern** (per point increase) (4669) |  | 0.82 (0.77 - 0.87) | <0.0001 | 0.67 (0.62 - 0.72) | <0.0001 |
| **Access to PPE** (4457) |  |  |  |  |  |
| Not all time | (Reference) | (Reference) |  | (Reference) |  |
| All the time |  | 0.61 (0.5 - 0.75) | <0.0001 | 0.47 (0.37 - 0.58) | <0.0001 |
| **Job redeployment** (4419) |  |  |  |  |  |
| No | (Reference) | (Reference) |  | (Reference) |  |
| Yes |  | 1.08 (0.91 - 1.28) | 0.40 | 1.47 (1.22 - 1.77) | <0.0001 |
| **Loneliness** (per point increase) (4903) |  | 1.12 (1.08 - 1.16) | <0.0001 | 1.24 (1.19 - 1.29) | <0.0001 |

**Supplementary Table 8: Relative risk ratios (95% CI) from multinomial logistic regression on imputed data, comparing membership of being in the very low attrition intention group compared to other group associated with covariates.**

| **Characteristics (N=5499)** | **Consistently Low** | **Moderate & increasing** | **P-value** | **Consistently high** | **P-value** |
| --- | --- | --- | --- | --- | --- |
| **Age Category** |  |  |  |  |  |
| 18-29 | (Reference) | (Reference) |  | (Reference) |  |
| 30-39 |  | 0.81 (0.64 -1.03) | 0.09 | 0.85 (0.63 -1.14) | 0.28 |
| 40-49 |  | 0.83 (0.65 -1.05) | 0.12 | 1.08 (0.8 -1.44) | 0.62 |
| 50-59 |  | 1.29 (1.02 -1.63) | 0.03 | 2.09 (1.58 -2.77) | <0.0001 |
| 60+ years |  | 1.17 (0.89 -1.52) | 0.26 | 1.56 (1.14 -2.14) | 0.01 |
| **Gender** |  |  |  |  |  |
| Male | (Reference) | (Reference) |  | (Reference) |  |
| Female |  | 1.10 (0.94 -1.28) | 0.25 | 1.16 (0.97 -1.38) | 0.11 |
| **Occupation** |  |  |  |  |  |
| Doctors and medical support | (Reference) | (Reference) |  | (Reference) |  |
| Nurses, NAs, Midwives |  | 1.28 (1.05 -1.57) | 0.02 | 1.39 (1.12 -1.73) | <0.0001 |
| AHPs (not including scientists) |  | 0.98 (0.82 -1.17) | 0.85 | 0.84 (0.69 -1.04) | 0.10 |
| Pharmacy |  | 1.08 (0.69 -1.7) | 0.73 | 0.53 (0.27 -1.03) | 0.06 |
| Healthcare scientist |  | 0.81 (0.58 -1.13) | 0.21 | 1.01 (0.71 -1.44) | 0.94 |
| Ambulance |  | 1.42 (0.99 -2.03) | 0.06 | 1.19 (0.78 -1.81) | 0.41 |
| Dental |  | 1.55 (1.16 -2.09) | <0.0001 | 1.79 (1.29 -2.48) | <0.0001 |
| Optical |  | 1.21 (0.79 -1.85) | 0.39 | 1.07 (0.65 -1.74) | 0.80 |
| Administrative |  | 0.86 (0.55 -1.35) | 0.51 | 0.56 (0.3 -1.03) | 0.06 |
| Other |  | 0.98 (0.68 -1.43) | 0.94 | 0.65 (0.39 -1.06) | 0.09 |
| **Ethnicity** |  |  |  |  |  |
| White British/Irish | (Reference) | (Reference) |  | (Reference) |  |
| White Other/GIT |  | 1.05 (0.82 -1.36) | 0.68 | 1.18 (0.89 -1.56) | 0.26 |
| Asian |  | 0.93 (0.77 -1.13) | 0.46 | 1.03 (0.82 -1.28) | 0.83 |
| Black |  | 0.57 (0.37 -0.88) | 0.01 | 0.67 (0.43 -1.04) | 0.07 |
| Mixed |  | 1.47 (1.08 -2.02) | 0.02 | 1.51 (1.05 -2.17) | 0.03 |
| Other |  | 1.13 (0.67 -1.92) | 0.64 | 0.72 (0.35 -1.48) | 0.37 |
| **Born abroad** |  |  |  |  |  |
| Born in UK | (Reference) | (Reference) |  | (Reference) |  |
| Born outside UK |  | 0.95 (0.81 -1.11) | 0.50 | 0.93 (0.77 -1.11) | 0.41 |
| **Financial Difficulties** (per point increase) |  | 1.25 (1.16 -1.34) | <0.0001 | 1.44 (1.33 -1.56) | <0.0001 |
| **Depression** |  |  |  |  |  |
| No | (Reference) | (Reference) |  | (Reference) |  |
| Yes |  | 1.61 (1.32 -1.97) | <0.0001 | 2.95 (2.38 -3.67) | <0.0001 |
| **Anxiety** |  |  |  |  |  |
| No | (Reference) | (Reference) |  | (Reference) |  |
| Yes |  | 1.65 (1.37 -1.99) | <0.0001 | 2.77 (2.28 -3.36) | <0.0001 |
| **PTSD** |  |  |  |  |  |
| No | (Reference) | (Reference) |  | (Reference) |  |
| Yes |  | 1.57 (1.37 -1.81) | <0.0001 | 2.24 (1.92 -2.62) | <0.0001 |
| **Patients attended** |  |  |  |  |  |
| Zero | (Reference) | (Reference) |  | (Reference) |  |
| Low |  | 1.02 (0.8 -1.31) | 0.87 | 0.96 (0.73 -1.26) | 0.75 |
| Medium |  | 1.17 (0.91 -1.5) | 0.22 | 1.02 (0.79 -1.33) | 0.86 |
| High |  | 1.44 (1.13 -1.83) | <0.0001 | 1.20 (0.91 -1.56) | 0.19 |
| **Discrimination** |  |  |  |  |  |
| No discrimination | (Reference) | (Reference) |  | (Reference) |  |
| From patients |  | 1.66 (1.37 -2.03) | <0.0001 | 1.85 (1.46 -2.34) | <0.0001 |
| From colleagues |  | 1.59 (1.25 -2.03) | <0.0001 | 2.56 (2.02 -3.26) | <0.0001 |
| From patients and colleagues |  | 2.07 (1.45 -2.95) | <0.0001 | 3.36 (2.38 -4.74) | <0.0001 |
| **Work Fairness** (per point increase) |  | 0.84 (0.77 -0.92) | <0.0001 | 0.71 (0.65 -0.78) | <0.0001 |
| **Feeling secure to raise concern** (per point increase) |  | 0.85 (0.79 -0.91) | <0.0001 | 0.76 (0.71 -0.82) | <0.0001 |
| **Trusting employer to address concern** (per point increase) |  | 0.83 (0.78 -0.89) | <0.0001 | 0.69 (0.65 -0.75) | <0.0001 |
| **Access to PPE** |  |  |  |  |  |
| Not all time | (Reference) | (Reference) |  | (Reference) |  |
| All the time |  | 0.65 (0.53 -0.79) | <0.0001 | 0.51 (0.42 -0.63) | <0.0001 |
| **Job redeployment** |  |  |  |  |  |
| No | (Reference) | (Reference) |  | (Reference) |  |
| Yes |  | 1.07 (0.89 -1.27) | 0.47 | 1.4 (1.17 -1.69) | <0.0001 |
| **Loneliness** (per point increase) |  | 1.12 (1.08 -1.16) | <0.0001 | 1.22 (1.17 -1.27) | <0.0001 |

**Supplementary Table 9: Relative risk ratios (95% CI) from multinomial logistic regression for consistently low vs. other attrition intention groups adjusted for all covariates.**

| **Characteristics (N=3269)** | **Consistently Low** | **Moderate & increasing** | **P-value** | **Consistently high** | **P-value** |
| --- | --- | --- | --- | --- | --- |
| **Age Category** |  |  |  |  |  |
| 18-29 | (Reference) | (Reference) |  | (Reference) |  |
| 30-39 |  | 0.99 (0.72 -1.35) | 0.94 | 1.07 (0.72 -1.6) | 0.74 |
| 40-49 |  | 1.05 (0.76 -1.43) | 0.78 | 1.63 (1.1 -2.4) | 0.01 |
| 50-59 |  | 1.94 (1.42 -2.64) | <0.0001 | 3.43 (2.34 - 5.04) | <0.0001 |
| 60+ years |  | 1.87 (1.28 -2.72) | <0.0001 | 2.56 (1.6 - 4.1) | <0.0001 |
| **Gender** |  |  |  |  |  |
| Male | (Reference) | (Reference) |  | (Reference) |  |
| Female |  | 1.14 (0.93 -1.39) | 0.22 | 1.10 (0.86 -1.4) | 0.45 |
| **Occupation** |  |  |  |  |  |
| Doctors and medical support | (Reference) | (Reference) |  | (Reference) |  |
| Nurses, NAs, Midwives |  | 1.15 (0.88 -1.49) | 0.30 | 1.02 (0.76 -1.38) | 0.89 |
| AHPs (not including scientists) |  | 0.99 (0.77 -1.27) | 0.94 | 0.72 (0.54 -0.97) | 0.03 |
| Pharmacy |  | 1.11 (0.63 -1.96) | 0.72 | 0.53 (0.23 -1.2) | 0.13 |
| Healthcare scientist |  | 0.93 (0.57 -1.52) | 0.78 | 0.87 (0.51 -1.5) | 0.63 |
| Ambulance |  | 1.04 (0.66 -1.64) | 0.86 | 0.71 (0.41 -1.22) | 0.21 |
| Dental |  | 1.56 (0.99 -2.46) | 0.05 | 1.77 (1.08 -2.91) | 0.03 |
| Optical |  | 1.68 (0.86 -3.29) | 0.13 | 0.61 (0.21 -1.71) | 0.35 |
| Administrative |  | 0.92 (0.48 -1.76) | 0.80 | 0.34 (0.13 -0.88) | 0.03 |
| Other |  | 0.94 (0.54 -1.65) | 0.84 | 0.55 (0.27 -1.15) | 0.11 |
| **Ethnicity** |  |  |  |  |  |
| White British/Irish | (Reference) | (Reference) |  | (Reference) |  |
| White Other/GIT |  | 1.13 (0.77 -1.65) | 0.54 | 1.23 (0.8 -1.9) | 0.35 |
| Asian |  | 0.79 (0.59 -1.06) | 0.12 | 0.60 (0.42 -0.86) | 0.01 |
| Black |  | 0.43 (0.24 -0.78) | 0.01 | 0.48 (0.25 -0.92) | 0.03 |
| Mixed |  | 1.02 (0.66 -1.58) | 0.94 | 1.17 (0.72 -1.91) | 0.52 |
| Other |  | 0.86 (0.42 -1.76) | 0.69 | 0.31 (0.11 -0.9) | 0.03 |
| **Born abroad** |  |  |  |  |  |
| Born in UK | (Reference) | (Reference) |  | (Reference) |  |
| Born outside UK |  | 0.82 (0.63 -1.08) | 0.16 | 0.85 (0.62 -1.17) | 0.32 |
| **Financial Difficulties** (per point increase) |  | 1.12 (1.01 -1.25) | 0.03 | 1.24 (1.1 -1.39) | <0.0001 |
| **Depression** |  |  |  |  |  |
| No | (Reference) | (Reference) |  | (Reference) |  |
| Yes |  | 1.02 (0.74 -1.41) | 0.89 | 1.59 (1.14 -2.24) | 0.01 |
| **Anxiety** |  |  |  |  |  |
| No | (Reference) | (Reference) |  | (Reference) |  |
| Yes |  | 1.26 (0.97 -1.64) | 0.09 | 1.33 (0.99 -1.79) | 0.06 |
| **PTSD** |  |  |  |  |  |
| No | (Reference) | (Reference) |  | (Reference) |  |
| Yes |  | 1.35 (1.11 -1.64) | <0.0001 | 1.59 (1.27 -1.99) | <0.0001 |
| **Patients attended** |  |  |  |  |  |
| Zero | (Reference) | (Reference) |  | (Reference) |  |
| Low |  | 1.12 (0.8 -1.58) | 0.51 | 1.08 (0.73 -1.6) | 0.69 |
| Medium |  | 1.19 (0.84 -1.68) | 0.34 | 1.00 (0.67 -1.49) | 1.00 |
| High |  | 1.53 (1.08 -2.16) | 0.02 | 1.12 (0.75 -1.66) | 0.59 |
| **Discrimination** |  |  |  |  |  |
| No discrimination | (Reference) | (Reference) |  | (Reference) |  |
| From patients |  | 1.52 (1.19 -1.95) | <0.0001 | 1.60 (1.2 -2.15) | <0.0001 |
| From colleagues |  | 1.16 (0.84 -1.6) | 0.38 | 1.55 (1.1 -2.18) | 0.01 |
| From patients and colleagues |  | 1.74 (1.11 -2.73) | 0.02 | 2.41 (1.5 -3.89) | <0.0001 |
| **Work Fairness** (per point increase) |  | 0.91 (0.8 -1.03) | 0.13 | 0.84 (0.73 -0.96) | 0.01 |
| **Feeling secure to raise concern** (per point increase) |  | 0.99 (0.87 -1.12) | 0.81 | 1.09 (0.95 -1.26) | 0.21 |
| **Trusting employer to address concern** (per point increase) |  | 0.89 (0.79 -1.00) | 0.05 | 0.76 (0.66 -0.86) | <0.0001 |
| **Access to PPE** |  |  |  |  |  |
| Not all time | (Reference) | (Reference) |  | (Reference) |  |
| All the time |  | 0.75 (0.58 -0.97) | 0.03 | 0.75 (0.56 -1.00) | 0.05 |
| **Job redeployment** |  |  |  |  |  |
| No | (Reference) | (Reference) |  | (Reference) |  |
| Yes |  | 1.03 (0.84 -1.26) | 0.80 | 1.32 (1.05 -1.66) | 0.02 |
| **Loneliness** (per point increase) |  | 1.02 (0.97 -1.08) | 0.39 | 1.04 (0.98 -1.1) | 0.22 |

References

1. Mésidor M, Rousseau M-C, O’Loughlin J and Sylvestre M-P. Does group-based trajectory modeling estimate spurious trajectories? *BMC Medical Research Methodology* 2022; 22: 194. DOI: 10.1186/s12874-022-01622-9.

2. Diop A, Gupta A, Mueller S, et al. Assessing the performance of group-based trajectory modeling method to discover different patterns of medication adherence. *Pharmaceutical Statistics* 2024; 23: 511-529. DOI: <https://doi.org/10.1002/pst.2365>.

3. Hickson RP, Annis IE, Killeya-Jones LA and Fang G. Opening the black box of the group-based trajectory modeling process to analyze medication adherence patterns: An example using real-world statin adherence data. *Pharmacoepidemiology and Drug Safety* 2020; 29: 357-362. DOI: 10.1002/pds.4917.

4. Hickson RP, Annis IE, Killeya-Jones LA and Fang G. Comparing Continuous and Binary Group-based Trajectory Modeling Using Statin Medication Adherence Data. *Medical Care* 2021; 59. DOI: 10.1097/MLR.0000000000001625.

5. Dara MS, Thomson WM, Jonathan MB, et al. High-risk glycated hemoglobin trajectories established by mid-20s: findings from a birth cohort study. *BMJ Open Diabetes Research & Care* 2016; 4: e000243. DOI: 10.1136/bmjdrc-2016-000243.
